# Performance of Elecsys Anti‐HEV IgG and IgM Assays for the Detection of Acute, Recent or Past Hepatitis E Virus Infection

**DOI:** 10.1111/liv.70480

**Published:** 2025-12-15

**Authors:** Mathias Schemmerer, Lisa Sophie Pflüger, Tanja Schneider, Jie Lu, Korbinian Kienle, Kristin Maria Meyer‐Schlinkmann, Annelies Mühlbacher, Johannes Polz, Harald Schennach, Qing Xie, Christian Voitenleitner, Jürgen Wenzel, Marc Lütgehetmann

**Affiliations:** ^1^ National Consultant Laboratory for HAV and HEV, Institute of Clinical Microbiology and Hygiene University Medical Center Regensburg Regensburg Germany; ^2^ Center for Diagnostics, Institute of Medical Microbiology, Virology and Hygiene University Medical Center Hamburg‐Eppendorf Hamburg Germany; ^3^ Roche Diagnostics GmbH Penzberg Germany; ^4^ Department of Infectious Diseases Ruijin Hospital, Shanghai Jiao Tong University School of Medicine Shanghai China; ^5^ MVZ Labor Krone eGbR, Studienzentrum Immunologie Bad Salzuflen Germany; ^6^ Central Institute for Blood Transfusion and Immunology, Tirol Kliniken GmbH Innsbruck Austria; ^7^ Roche Diagnostics International AG Rotkreuz Switzerland; ^8^ German Center for Infection Research (DZIF), Hamburg‐Lübeck‐Borstel‐Riems Site Hamburg Germany

**Keywords:** blood donors, hepatitis antibodies, hepatitis E virus, seroconversion, viremia

## Abstract

**Background & Aims:**

Hepatitis E virus (HEV) is the leading cause of acute viral hepatitis. We evaluated the performance of the new automated Elecsys Anti‐HEV IgG and Elecsys Anti‐HEV IgM assays (Roche Diagnostics) in detecting acute, recent and past HEV infection.

**Methods:**

Performance of the Elecsys assays relative to three commercially available CE‐IVD anti‐HEV assays was assessed in a large multinational cohort (six laboratories in Germany, Austria and China). Sensitivity testing included samples from patients with presumed acute (IgM: *n* = 707; IgG: *n* = 490) and recovered HEV infection (*n* = 156). Specificity testing included samples from asymptomatic blood donors (*n* = 5040), routine diagnostic samples (*n* = 2427), and pregnant women (*n* = 544). An in‐house anti‐HEV IgG neutralisation method was performed to resolve discrepant specificity sample results. Seroconversion sensitivity analysis was performed using nine commercial seroconversion panels (*n* = 119 samples).

**Results:**

Relative sensitivity and specificity of the Elecsys assays were > 98.6% (95% CI: 92.2%–100%) and > 98.9% (98.4%–99.2%) for anti‐HEV IgM and > 81.2% (79.3%–83.0%) for anti‐HEV IgG in different cohorts, respectively. After confirming anti‐HEV IgG in 680/696 discrepant samples by a neutralisation method, relative specificity of the Elecsys Anti‐HEV IgG assay was > 99.4% (98.8%–99.7%). Evaluation of commercial seroconversion panels showed good overall agreement between the assays.

**Conclusions:**

The Elecsys Anti‐HEV IgG and IgM assays showed favourable overall performance when compared to three commercially available CE‐IVD marked assays. Neutralisation data indicated higher sensitivity of the Elecsys Anti‐HEV IgG assay than comparator assays in detecting past infections and lower IgG concentrations.

## Lay Summary

Our study was conducted at six laboratories in Germany, Austria and China. We evaluated the performance of two new tests (Elecysys Anti‐HEV IgG and IgM assays [Roche Diagnostics]) by measuring how well they can identify hepatitis E virus in people who have been infected—which is known as sensitivity. Our results suggest that these tests are more sensitive in detecting previous hepatitis E infections than some other tests, which are already available in Europe.

**Abbreviations**
CI: confidence interval
COI: cutoff index
ELISA: enzyme‐linked immunosorbent assay
HEV: hepatitis E virus
IgG: immunoglobulin G
IgM: immunoglobulin M
OPA: overall percentage agreement
PCR: polymerase chain reaction
WHO: World Health Organization

## 1 Introduction

An estimated 900 million people, or ~1/8 of the global population, have been exposed to hepatitis E virus (HEV, *Paslahepevirus balayani*), and 15–110 million people have recent/ongoing infection [1, 2]. HEV comprises eight genotypes (HEV‐1 to ‐8), of which four (HEV‐1 to ‐4) are mainly responsible for infections in humans [3, 4]. HEV‐1 and HEV‐2 variants are transmitted through drinking water and account for about 3 million symptomatic cases annually in the tropics [4, 5, 6]; HEV‐3 and HEV‐4 infections are reported in developed countries and are transmitted zoonotically by direct contact with infected animals, or by eating contaminated meat [4, 6]. HEV‐5, to ‐8 infect animals, and only one case of HEV‐7 infection in a human has been described to date [3, 5, 6]. HEV may also be transmitted by blood transfusion and by organ transplantation; however, recipients have a higher risk of acquiring HEV infection from diet than from transplantation [7, 8, 9]. Furthermore, this patient cohort is also prone to infection from another species within the *Hepeviridae* family—rat HEV (*Rocahepevirus ratti*)—for which, 37 cases have been documented in humans worldwide since 2018 [10, 11]. Clinically, most HEV infections are asymptomatic or self‐limiting with only mild symptoms [5, 12]. However, infections can also cause severe disease with acute or acute‐on‐chronic liver failure, obstetric complications (HEV‐1 and ‐2) and chronic infection in severely immunosuppressed patients (HEV‐3 and ‐4) [4, 6]. After HEV exposure, the incubation period is typically 5–6 weeks, with HEV RNA detectable in serum for roughly 45–101 days after infection, depending on the limit of detection of the molecular assay [2, 4]. In immunocompetent patients, during acute infection, anti‐HEV IgM antibodies are detectable with the onset of clinical symptoms, such as nausea, vomiting, diarrhoea, abdominal pain, fever, jaundice and elevation of serum transaminase; anti‐HEV IgG antibodies are detectable later and persist for a longer duration [4, 5, 12].

For the diagnosis of acute HEV infection, the European Association for the Study of the Liver recommends assessing HEV RNA alone or in combination with anti‐HEV IgG and/or anti‐HEV IgM [13]. Acute infection in immunocompetent people may also be diagnosed by testing for anti‐HEV IgM in combination with anti‐HEV IgG; however, HEV RNA testing is essential for those who are immunocompromised [13]. HEV RNA testing is routinely used in several European countries to screen blood donations [9]. In 2023, serological testing for IgM was added as a means of diagnosing acute HEV infection in the World Health Organization's (WHO) Essential Diagnostics List [14].

Commonly used HEV serology assays include enzyme‐linked immunosorbent assays (ELISAs) and automated‐based chemiluminescence assays. However, large differences in clinical performance across different anti‐HEV IgM and IgG assays represent a challenge for the surveillance of HEV exposure and mitigate HEV infections in vulnerable populations [1, 5, 10, 15, 16]. Additionally, many serological tests are not fully automated and are more time‐intensive than fully automated assays. The Elecsys Anti‐HEV IgG and Elecsys Anti‐HEV IgM assays are analysed on the Cobas e system, which is a widely used fully automated analysis platform providing short time‐to‐result and on‐demand processing.

The aim of this study was to evaluate the performance of two new Elecsys assays for the detection of anti‐HEV IgM and anti‐HEV IgG in samples from a large multinational cohort.

## 2 Methods

### 2.1 Study Design

**FIGURE 1:**
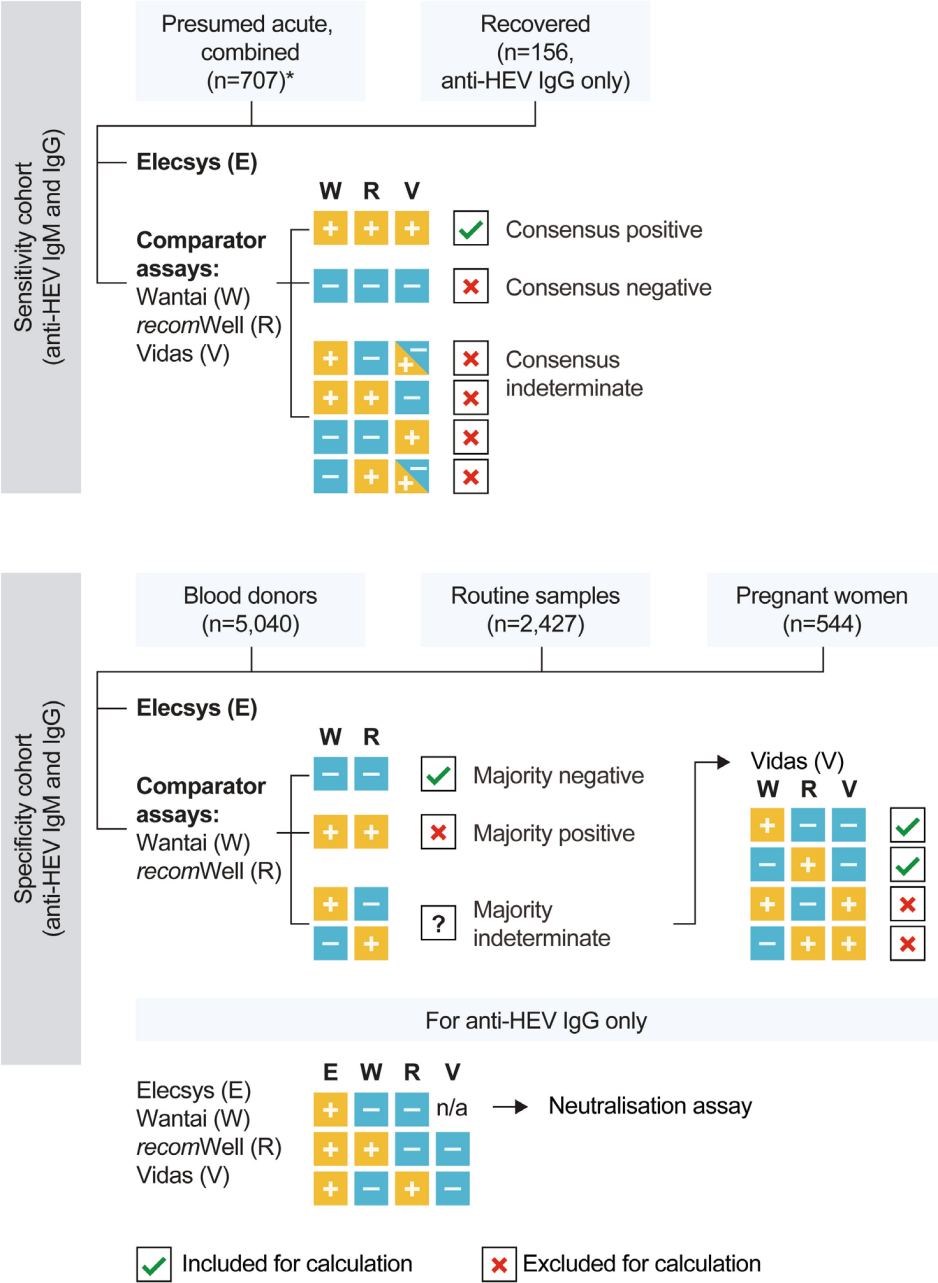
Study design overview. *Combined means ‘presumed acute IgM‐positive’ cohort and ‘HEV PCR‐positive’ cohort. E, Elecsys; HEV, hepatitis E virus; R, *recom*Well; V, Vidas; W, Wantai.

An overview of the study design is shown in Figure 1. The study was performed between March 2022 and January 2023. Sensitivity and specificity of the Elecsys Anti‐HEV IgG and Elecsys Anti‐HEV IgM assays, relative to CE‐IVD commercial assays, were assessed at six independent diagnostic laboratories (Germany, *n* = 4; Austria, *n* = 1; China, *n* = 1; Table S1). Details of ethics approval and samples tested, according to study site, are provided in Table S2. The study was performed according to the ethical principles originating in the Declaration of Helsinki. Seroconversion and titre development assessments were performed by Roche Diagnostics GmbH, Penzberg (Germany).

### 2.2 Study Population

The sensitivity evaluation was conducted with samples from individuals with presumed acute HEV infection or presumed recovered HEV infection with a pretested IgG positive result (only relevant for IgG).

Samples were categorised as ‘presumed acute HEV infection’ if they were pre‐tested reactive for anti‐HEV IgM in a routine assay, and as ‘HEV PCR‐positive’ if they yielded a positive result in the routine HEV‐PCR test.

The specificity evaluation was conducted with samples from a healthy population of blood donors, routine samples for hepatitis testing and pregnant women. Cohorts included samples from regions endemic for HEV‐1, ‐3 and ‐4; a subset of samples from individuals with presumed acute HEV infection were genotyped at two study sites: Regensburg (Germany) and Shanghai (China). All samples were anonymised/pseudonymised residual diagnostic samples.

### 2.3 Elecsys Anti‐HEV IgG and Elecsys Anti‐HEV IgM Assays

The Elecsys Anti‐HEV IgM and Anti‐HEV IgG assays are automated electrochemiluminescence immunoassays for use on Cobas e immunoassay analysers that enable the qualitative (IgM) and qualitative/quantitative (IgG) determination of anti‐HEV antibodies. Assays were performed according to the manufacturer's instructions, with a time to result of 18 min.

The Elecsys Anti‐HEV IgM assay is based on the μ‐Capture test principle [17]. For the first incubation, 6 μL (Cobas e 402 and e 801) or 10 μL (Cobas e 411, e 601 and e 602) of sample were automatically prediluted 1:20 with Diluent Universal. Ruthenium‐labelled HEV recombinant antigen reacted with anti‐HEV IgM antibodies in the sample. Biotinylated monoclonal human IgM‐specific antibodies and streptavidin‐coated microparticles were then added. After the immune complex bound to the solid phase via the interaction of biotin and streptavidin, the mixture was aspirated into a measuring cell to assess voltage‐induced chemiluminescence. Samples with a cutoff index (COI) of ≥ 1.0 and < 1.0 were reactive and non‐reactive (i.e., anti‐HEV IgM‐positive and ‐negative), respectively.

The Elecsys Anti‐HEV IgG assay is based on a double antigen sandwich principle. For the first incubation, 12 μL (Cobas e 402 and e 801) or 20 μL (Cobas e 411, e 601 and e 602) of sample was added to biotinylated HEV recombinant antigen, designed for the detection of anti‐HEV IgG and ruthenium‐labelled HEV recombinant antigen, forming a sandwich immune complex. Streptavidin‐coated microparticles were added, facilitating binding of the immune complex to the solid phase via interaction of biotin and streptavidin. The mixture was aspirated into a measuring cell and voltage‐induced chemiluminescence measured. The assay was standardised against WHO anti‐HEV reference reagent (95/584) [18]. The measuring range of the Elecsys Anti‐HEV IgG assay for undiluted samples was 0.05–25 U/mL and could be extended by diluting the samples accordingly (e.g., up to 2500 U/mL for 100‐fold diluted samples). The Elecsys Anti‐HEV IgG assay could also be qualitatively interpreted for the detection of IgG antibodies to HEV. Samples with an anti‐IgG concentration of ≥ 0.15 U/mL were considered reactive for anti‐HEV IgG; samples with a concentration of < 0.15 U/mL considered non‐reactive for anti‐HEV IgG.

### 2.4 Comparator Antibody Assays

CE‐IVD approved comparator assays used in this study included: *recom*Well HEV IgG/IgM (Mikrogen Diagnostik, Neuried, Germany), Wantai Anti‐HEV IgM/Anti‐HEV IgG (Wantai BioPharm, Beijing, China), Vidas Anti‐HEV IgM/Anti‐HEV IgG (bioMérieux Ltd., Basingstoke, UK) and Wantai InnoDx HEV IgM/IgG (Wantai BioPharm, Beijing, China). An overview of the comparator assays used is provided in Table S3. The assays were performed according to the manufacturer's instructions, including retesting of borderline results. Samples from China that were included in the sensitivity cohort were measured with three anti‐HEV IgG assays available in China (i.e., *recom*Well, Wantai ELISA and Wantai InnoDx).

### 2.5 Relative Sensitivity

The sensitivity of the Elecsys Anti‐HEV IgM and IgG assays was assessed relative to the Wantai, *recom*Well and Vidas assays, using a consensus approach—a strategy that represents the gold standard for detection of anti‐HEV IgG and IgM, due to varying performance of competitor assays. Samples were considered positive if results for anti‐HEV IgM or anti‐HEV IgG were positive with all three comparator assays. For genotype 4 samples, the sensitivity was determined relative to the consensus approach of Wantai, *recom*Well and InnoDx assays.

### 2.6 Relative Specificity

The relative specificity of the Elecsys Anti‐HEV IgM and IgG assays was assessed relative to the Wantai, *recom*Well and Vidas assays using a majority approach, whereby samples were considered negative for anti‐HEV IgM or anti‐HEV IgG if negative results were observed in two‐thirds of comparator assays.

### 2.7 Anti‐HEV IgG Resolution Testing by Neutralisation

Specificity samples with a discrepant reactive Elecsys Anti‐HEV IgG result, compared to the majority of comparator tests, were retested using an in‐house neutralisation method similar to that reported previously [19, 20]. Briefly, a preincubation with a recombinant HEV antigen was performed and compared to a reference (same volume of anti‐HEV IgG added). If the recovery was ≤ 50%, the sample was considered confirmed‐reactive (i.e., a true positive result). If the recovery was > 50%, the sample was considered false‐reactive with the Elecsys Anti‐HEV IgG assay. See the Supporting Information for a detailed description of the method and validation.

### 2.8 Seroconversion Sensitivity Analysis

The seroconversion sensitivity of Elecsys Anti‐HEV IgG and IgM assays was assessed using nine commercially available HEV seroconversion panels (Panels A–I; BIOMEX GmbH, Heidelberg, Germany) [21], and results were compared with those from *recom*Well, Wantai and Vidas assays. Results from *recom*Well HEV IgG and HEV polymerase chain reaction (PCR) assays were used as recommended on the package insert of the BIOMEX panels [21].

### 2.9 Statistics

Relative sensitivity and relative specificity for the Elecsys Anti‐HEV IgM and Elecsys Anti‐HEV IgG, and the combined comparator results, were calculated as follows:

$$\text{Relative sensitivity}=\frac{\text{congruent positive}\;\left(\text{Elecsys};\text{consensus comparator tests}\right)}{\text{positive}\;\left(\text{consensus comparator tests}\right)}$$

$$\text{Relative specificity}=\frac{\text{congruent positive}\;\left(\text{Elecsys};\text{Majority}\right)}{\text{positive}\;\left(\text{Majority}\right)}$$

The overall percentage agreement (OPA) between the Elecsys Anti‐HEV IgM or IgG assay and comparator assays was calculated as follows:

$$\mathrm{OPA}=\frac{\text{concordant positive}+\text{concordant negative}}{\text{concordant positive}+\text{concordant negative}+\text{disconcordant positive}+\text{discordant negative}}$$

The distribution of results of sensitivity and specificity assessments were reported according to COI increments for Elecsys Anti‐HEV IgM and U/mL increments for Elecsys Anti‐HEV IgG; results were presented in parallel with consensus reactive results (sensitivity cohort) and majority non‐reactive results (specificity cohort), including re‐analysed discrepant results.

Violin plots were developed representing anti‐HEV IgM and anti‐HEV IgG levels in the sensitivity cohort. Results for anti‐HEV IgM and IgG assays were log_10_ converted and, dependent on calibration results, presented in COI, A/CO or U/mL for all samples (total) and according to presumed HEV genotype (HEV‐1, ‐3 and ‐4).

The analysis was programmed using R or SAS. The 95% two‐sided confidence intervals (CIs) were reported using the Clopper–Pearson exact method.

## 3 Results

### 3.1 Relative Sensitivity

The sensitivity assessment for Elecsys Anti‐HEV IgM included 707 predetermined samples from individuals with presumed acute HEV infection (*n* = 490) and HEV RNA PCR‐positive samples (*n* = 217). The individuals were from Europe where HEV‐3 is endemic (*n* = 469), from Vietnam and Bangladesh where HEV‐1 is endemic (*n* = 188), and from China where HEV‐4 is endemic (*n* = 50; Table S2). The Elecsys Anti‐HEV‐IgG sensitivity assessment included 863 predetermined samples from individuals with presumed acute HEV infection (*n* = 490), samples with HEV PCR‐positive results (*n* = 217), and samples from European individuals with recovered HEV infection (*n* = 156; Table S2). A visual representation of the sensitivity cohort with the different assays is provided in Figure 1.

#### 3.1.1 Anti‐HEV IgM

In the presumed acute HEV infection cohort (*n* = 440), 359 samples (without HEV‐4 samples) tested anti‐HEV IgM‐positive in all three comparator assays (consensus approach) and were considered in the sensitivity calculation. The remaining samples had negative (*n* = 37) or indeterminate results (*n* = 43); one sample was excluded due to missing Elecsys Anti‐HEV‐IgM result. The relative sensitivity of the Elecsys Anti‐HEV IgM assay for the presumed acute cohort was 98.6% (95% CI: 96.8–99.5; Table 1).

**TABLE 1:** Sensitivity of Elecsys Anti‐HEV IgM and IgG assays relative to combined comparator assay results.

Cohort	Positive results comparator assay^a^	Positive Elecsys	Relative sensitivity
	*n*	*N*	% (95% CI)
Anti‐HEV IgM			
Presumed acute (*n* = 440)^b^	359	354	98.6 (96.8–99.5)
Presumed acute (China, HEV‐4; *n* = 50)	49	49	100 (92.7–100)
Anti‐HEV IgG			
Presumed acute (*n* = 440)	380	375	98.7 (97.0–99.6)
Presumed acute (China, HEV‐4; *n* = 50)	48	48	100 (92.6–100)
Recovered (*n* = 156)	141	141	100 (97.4–100)

Abbreviations: CI, confidence interval; HEV, hepatitis E virus; Ig, immunoglobulin; PCR, polymerase chain reaction.

^a^ Positive by three comparator assays (Wantai, *recom*Well and Vidas); in China, the comparator assays were Wantai ELISA, *recom*Well and Wantai InnoDx.

^b^ Analysis excluded one sample due to a missing Elecsys HEV IgM result.

In the PCR‐positive sample cohort (*n* = 217), 69 samples tested anti‐HEV IgM‐positive in all three comparator assays (consensus approach) and were considered in the sensitivity calculation. The remaining samples had negative (*n* = 128) or indeterminate results (*n* = 17); three samples were excluded due to missing Elecsys Anti‐HEV‐IgM results. The relative sensitivity of the Elecsys Anti‐HEV IgM assay for the PCR‐positive cohort was 98.6% (95% CI: 92.2–100).

The relative sensitivity of the Elecsys Anti‐HEV IgM assay, compared with the consensus of the Wantai, *recom*Well and InnoDx assays, for acute HEV‐4 infection was 100.0% (95% CI: 92.7–100; Table 1).

#### 3.1.2 Anti‐HEV IgG

Among the 440 presumed acute samples (without HEV‐4 samples), 380 showed a concordant positive anti‐HEV IgG result in all three comparator tests, 33 were negative and 27 were indeterminate. The relative sensitivity of the Elecsys Anti‐HEV IgG assay for the presumed acute cohort was 98.7% (95% CI: 97.0–99.6; Table 1). In the HEV PCR‐positive sample cohort (*n* = 217), 83 samples tested anti‐HEV IgG‐positive in all three comparator assays (consensus approach) and were considered in the sensitivity calculation. The remaining samples had negative (*n* = 123) or indeterminate results (*n* = 11). The relative sensitivity of the Elecsys Anti‐HEV IgG assay for the HEV PCR‐positive cohort was 91.6% (95% CI: 83.4–96.5). Among samples with presumed acute HEV‐4 infection, the relative sensitivity of the Elecsys assay compared to the consensus of Wantai, *recom*Well and InnoDx assays was 100.0% (95% CI: 92.6–100; Table 1).

Among the presumed recovered sample cohort, 141 were anti‐HEV IgG‐positive, 7 were anti‐HEV IgG‐negative and 8 samples were indeterminate. The relative sensitivity of the Elecsys Anti‐HEV IgG assay for this cohort was 100% (95% CI: 97.4–100; Table 1).

### 3.2 Relative Specificity

In total, 8011 samples were assessed, all from Europe. A visual representation of the specificity cohort from the different assays is provided in Figure 1.

#### 3.2.1 Anti‐HEV IgM

Using the majority approach on the 5040 blood donor samples, 4995 had a negative result, 39 were positive and 4 were indeterminate. Two samples were excluded due to missing Elecsys Anti‐HEV‐IgM results. The relative specificity of the Elecsys Anti‐HEV IgM assay was 99.6% (95% CI: 99.4–99.8; Table 2).

**TABLE 2:** Specificity of Elecsys Anti‐HEV IgM and IgG assays relative to combined comparator assay result.

Cohort	Negative comparator assay^a^	Negative Elecsys	Remaining negative after neutralisation	Relative specificity before neutralisation	Relative specificity after neutralisation
	*n*	*n*	*n*	% (95% CI)	% (95% CI)
Anti‐HEV IgM					
Blood donors (*n* = 5040)^b^	4995	4977	n/a	99.6 (99.4–99.8)	n/a
Routine samples (*n* = 2427)	2375	2348	n/a	98.9 (98.4–99.2)	n/a
Pregnant women (*n* = 544)	531	531	n/a	100 (99.3–100)	n/a
Anti‐HEV IgG					
Blood donors (*n* = 5040)	4397	4051	4055	92.1 (91.3–92.9)	99.9 (99.7–100)
Routine samples (*n* = 2427)	1754	1425	1434	81.2 (79.3–83.0)	99.4 (98.8–99.7)
Pregnant women (*n* = 544)	455	434	435	95.4 (93.0–97.1)	99.8 (98.7–100)

Abbreviations: CI, confidence interval; HEV, hepatitis E virus; Ig, immunoglobulin.

^a^ Negative by at least two of three comparator assays: Wantai assay, *recom*Well assay and Vidas assay.

^b^ Analysis excluded two samples due to missing Elecsys HEV IgM result.

For anti‐HEV IgM detection in samples from routine diagnostics, 2375/2427 samples were negative, 50 were positive and 2 were indeterminate. The relative specificity of the Elecsys Anti‐HEV IgM assay in routine samples was 98.9% (95% CI: 98.4–99.2; Table 2).

Among the pregnant women group, 531/544 samples were negative, 12 were positive and 1 sample was indeterminate. The relative specificity of the Elecsys Anti‐HEV IgM assay in this cohort was 100% (95% CI: 99.3–100; Table 2).

#### 3.2.2 Anti‐HEV IgG

Using the majority approach on the 5040 blood donor samples, 4397 were anti‐HEV IgG‐negative, 622 were positive and 21 were indeterminate. The initial relative specificity of the Elecsys Anti‐HEV IgG assay was 92.1% (95% CI: 91.3–92.9). Of the 346 discrepant samples, 344 were further analysed by the neutralisation protocol, which showed 340 samples to have true‐positive results. The adjusted relative specificity for the blood donor cohort, using the re‐analysed data, was 99.9% (95% CI: 99.7–100; Table 2).

Among the routine diagnostics cohort, 1754/2427 samples were anti‐HEV IgG‐negative, 655 were positive and 18 were indeterminate. The initial relative specificity of the Elecsys Anti‐HEV IgG assay in this group was 81.2% (95% CI: 79.3–83.0). Re‐analysis of the 329 samples with discrepant positive Elecsys Anti‐HEV IgG results showed that 320 samples had true‐positive results. Adjusting for the reanalysed data, the relative specificity for the routine sample cohort was 99.4% (95% CI: 98.8–99.7; Table 2).

Among the cohort of 544 pregnant women, 455 samples were anti‐HEV IgG‐negative, 88 were positive and 1 was indeterminate. The initial relative specificity of the Elecsys Anti‐HEV IgG assay in this cohort was 95.4% (95% CI: 93.0–97.1). Of the 21 samples with a discrepant positive Elecsys Anti‐HEV IgG test, 20 had confirmed‐positive results using the neutralisation method, resulting in an adjusted relative specificity of 99.8% (95% CI: 98.7–100; Table 2).

### 3.3 Overall Percentage Agreement

**FIGURE 2:**
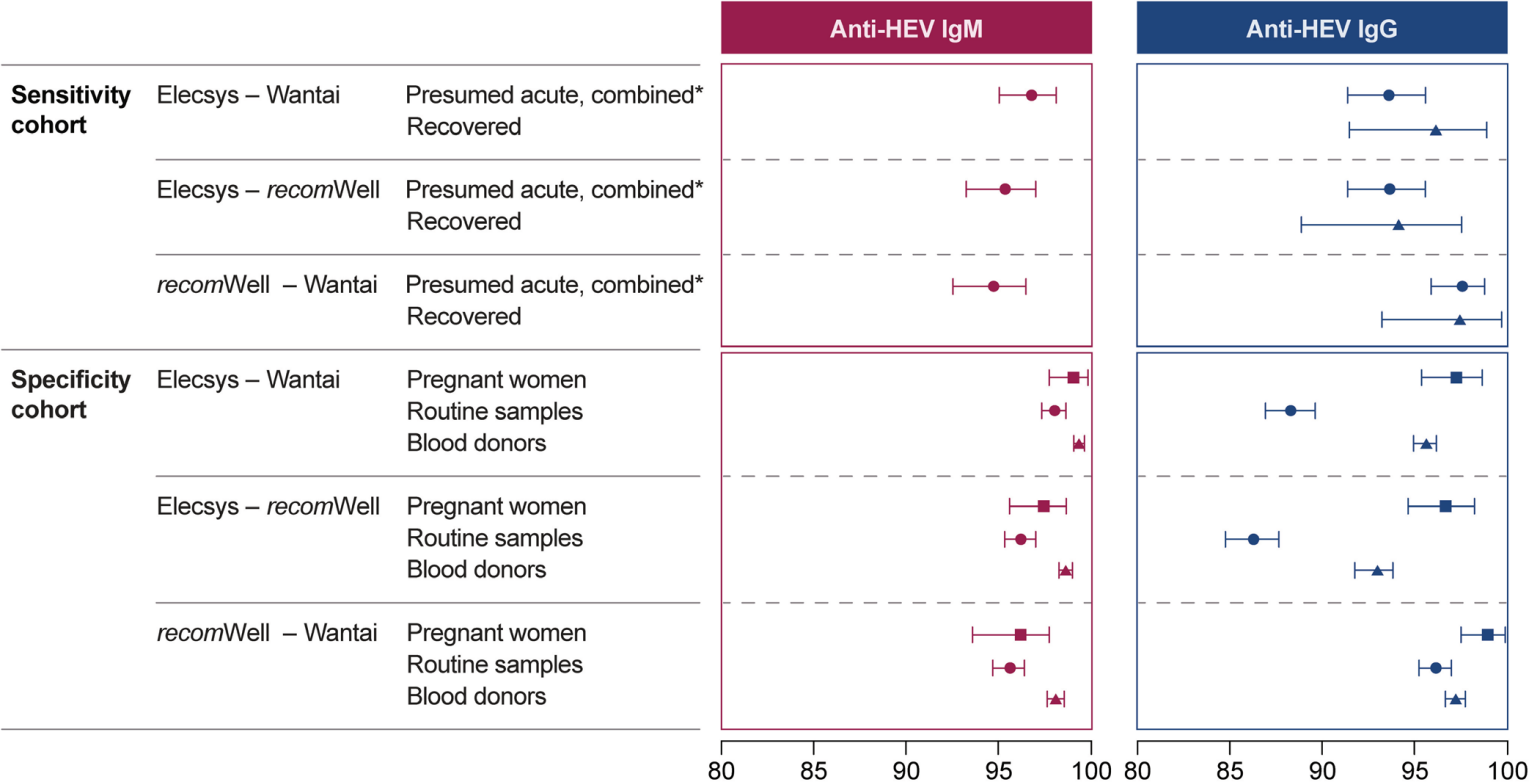
OPA between the Elecsys Anti‐HEV IgM or the Elecsys Anti‐HEV IgG assay and comparator assays for the sensitivity and specificity cohorts. *Combined means ‘presumed acute HEV infection’ cohort and ‘HEV PCR‐positive’ cohort. HEV, hepatitis E virus; OPA, overall percentage agreement.

The OPA between the Elecsys Anti‐HEV IgM and IgG assays and the Wantai and *recom*Well comparator assays showed that there was high agreement rates and overlapping CIs for all specificity and sensitivity cohorts (Figure 2).

Figure S1 shows the results for Elecsys Anti‐HEV IgM and IgG, and the comparator assays, grouped according to the assay result (reactive, non‐reactive, indeterminate/borderline).

### 3.4 Distribution of Elecsys Anti‐HEV IgM and IgG Results

**FIGURE 3:**
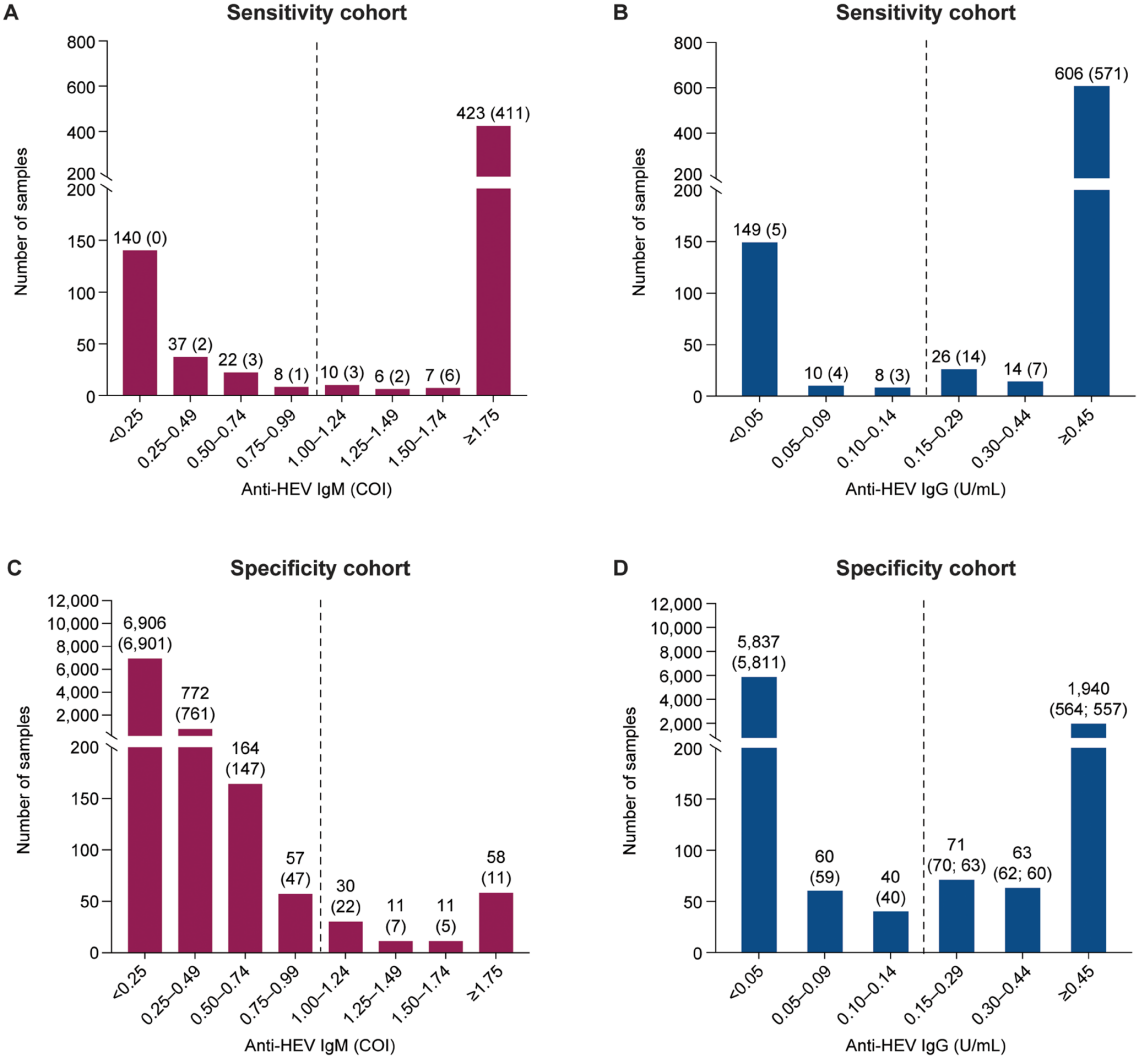
Distribution of test results around the cutoff (dashed line) for the Elecsys Anti‐HEV IgM (dark red; COI) and Anti‐HEV IgG (dark blue; U/mL). (A, B) For the sensitivity cohort (HEV‐1 and ‐3), the values in parentheses in each increment represent the number of samples achieving a consensus reactive result with the comparator assays. (C, D) For the specificity cohort, values in parentheses represent the number of samples achieving a majority non‐reactive result with the comparator assays. In (D), the second number in parentheses for the 0.15–0.29, 0.30–0.44 and ≥ 0.45 U/mL intervals represents the number of discrepant samples^†^ that were confirmed to have true‐positive Elecsys Anti‐HEV IgG results by neutralisation. ^†^Samples that yielded a discrepant reactive Elecsys Anti‐HEV IgG test result (compared with the majority‐negative result with comparator assays) were re‐analysed using an in‐house neutralisation method to confirm if Elecsys Anti‐HEV IgG‐positive results represented a true‐positive or a false‐positive results. COI, cut‐off index; HEV, hepatitis E virus.

The distribution of Elecsys Anti‐HEV IgM and IgG results, according to COI or U/mL values, for all included samples from the sensitivity cohort (HEV‐IgM: HEV‐PCR‐positive: *n* = 214; presumed acute without HEV‐4: *n* = 439; HEV‐IgG: HEV‐PCR‐ positive: *n* = 217; presumed acute without HEV‐4: *n* = 440; recovered: *n* = 156; Table 1) and all included samples from the specificity cohort (HEV‐IgM: *n* = 8009; HEV‐IgG: *n* = 8011; Table 2) is shown in Figure 3. The number in brackets per interval represents the comparator assay results (consensus reactive for the sensitivity cohort and majority non‐reactive for the specificity cohort). The second number in brackets for the 0.15–0.29, 0.30–0.44 and ≥ 0.45 U/mL intervals (Figure 3D) represents the number of discrepant samples that were confirmed to be true‐positive results by neutralisation.

The Elecsys Anti‐HEV IgM and IgG assays revealed good discrimination between reactive and non‐reactive samples. A small number of samples were found to have COI or U/mL values around cutoff (Anti‐HEV IgM: *n* = 18/653 [sensitivity], *n* = 87/8009 [specificity]) with values ranging from 0.75 to 1.24 COI; Anti‐HEV IgG: *n* = 34/813 (sensitivity), *n* = 111/8011 (specificity) with values ranging from 0.10 to 0.29 U/mL.

**FIGURE 4:**
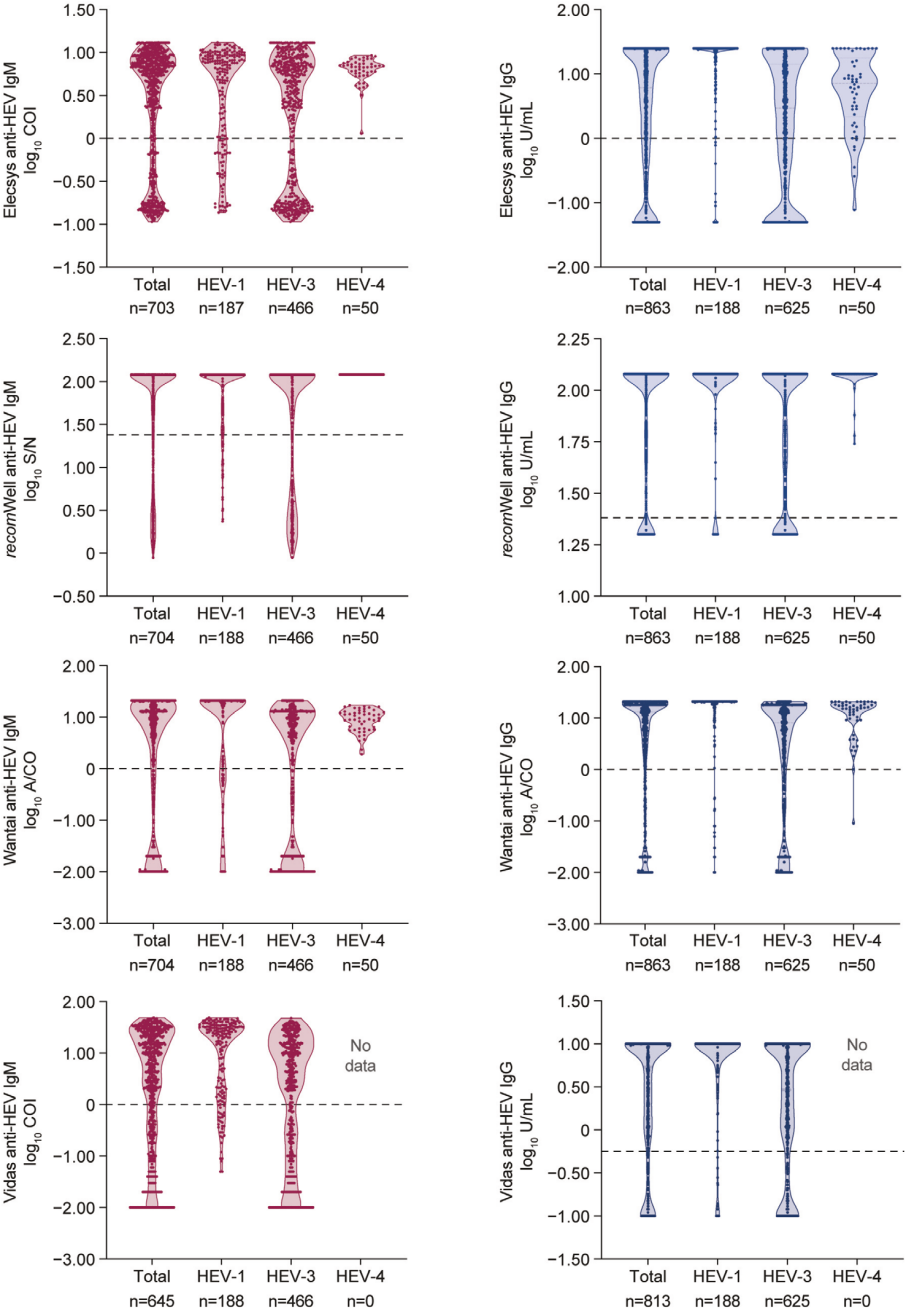
Violin plots of anti‐HEV IgM (dark red) and anti‐HEV IgG (dark blue) levels (total and by genotype) in the sensitivity cohort, according to the different anti‐HEV IgM and IgG assays, in samples from individuals with valid results for IgM (included the presumed acute HEV infection and the HEV‐PCR positive cohorts) and IgG (included presumed HEV acute infection, HEV‐PCR positive and recovered HEV infection cohorts). Samples were tested with Elecsys, *recom*Well, Wantai and Vidas IgM and IgG assays and dependent on calibration results were depicted on a log_10_ scale in COI, A/CO, or U/mL. Results are presented for all samples (total) and according to presumed HEV genotype (HEV‐1, ‐3 and ‐4). A/CO, appropriate cut‐off; COI, cut‐off index; HEV, hepatitis E virus.

Figure 4 shows that the individual results of the presumed acute cohort samples were distributed over the entire measuring range of the four anti‐HEV IgM and IgG assays and that all genotypes could be detected without any differences between the assays.

### 3.5 Seroconversion Sensitivity

**FIGURE 5:**
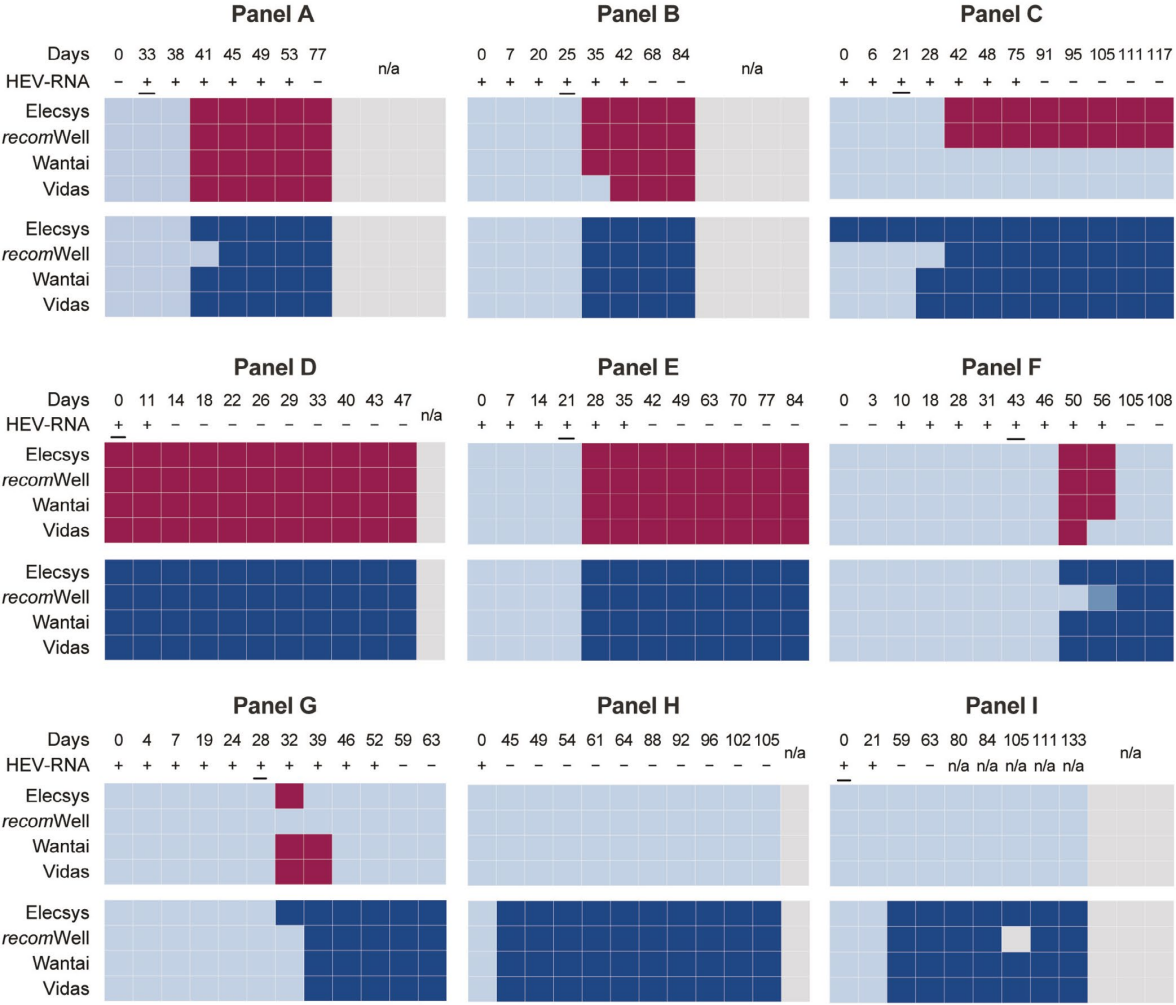
Sensitivity of the Elecsys assays and comparator assays for the detection of anti‐HEV IgM (dark red) and IgG (dark blue) in seroconversion panels. Seroconversion sensitivity was tested using nine commercial panels (A–I); samples (*N* = 119) were tested with Elecsys IgM and IgG, *recom*Well IgM (values from *recom*Well IgG were transferred from IFU), and Wantai and Vidas IgM and IgG assays. The days after first bleed, the *recom*Well IgG results, and the HEV quantitative PCR results were taken from the vendor description. Underlined timepoints represent HEV viral RNA peak. HEV, hepatitis E virus; IFU, instructions for use.

Seroconversion sensitivity analysis showed similar results for Panels A and E with either Elecsys Anti‐HEV IgM or with the three comparator tests; results were positive 41 days (panel A) and 28 days (panel E) after first bleed (Figure 5). Panels B and F showed similar results with Elecsys, *recom*Well and Wantai, with positivity at 35–84 and 50–56 days after first bleed, respectively, while Vidas showed positivity at 42–84 and 50 days after first bleed. For panel C, only Elecsys Anti‐HEV and *recom*Well showed similar seroconversion sensitivity, whereas Wantai and Vidas did not show any reactivity. Panel G showed a positive IgM result 32 days after first bleed with Elecsys, Wantai and Vidas; both comparator tests were also positive at 39 days, while *recom*Well was negative throughout. Panel D was positive from the first timepoint available with all assays. Panels H and I showed no reactivity through all bleeds with any of the assays (Figure 5).

Similar results with panels B, E, H and I were observed for Elecsys Anti‐HEV IgG and the three comparator tests, with reactivity 35 days (panel B), 28 days (panel E), 45 days (panel H) or 59 days (panel I) after the first bleed (Figure 5). In panels A and F, *recom*Well showed reactivity one bleed later compared to Elecsys and the other two comparator tests. Panel D was positive from the first timepoint available with all assays. The Elecsys IgG assay showed reactivity for panel C from the first bleed, while the comparator tests showed a positive result several blood draws later (Day 28: Vidas, Wantai; Day 42: *recom*Well). Elecsys Anti‐HEV IgG also showed a reactive panel G result earlier (Day 32) than the comparator tests (Day 39; Figure 5). In all nine panels (A–I), quantitative anti‐HEV IgG levels increased over time up to a maximum of 101 U/mL (Day 42 for panel C; Table S4).

## 4 Discussion

In this study, we evaluated two newly developed automated Elecsys assays for the detection of anti‐HEV IgM and anti‐HEV IgG in comparison to three widely used commercially available assays in a large multinational cohort and using nine seroconversion panels. Our results show that both Elecsys assays have good overall analytic performance, with high relative sensitivities and specificities.

The relative sensitivity was first assessed using a consensus approach, whereby samples with presumed acute HEV infection were considered positive if all three comparator assays had congruently detected anti‐HEV antibodies. Using this approach, the relative sensitivity of Elecsys Anti‐HEV IgM was 98.6%, 98.6% and 100% in presumed acute HEV‐infected (*n* = 440), HEV PCR positive (*n* = 217) and presumed HEV‐4 acute‐infected (*n* = 50) individuals, respectively. The relative sensitivity of Elecsys Anti‐HEV IgG was high at 98.7%, 100%, 91.6% and 100% in individuals presumed to have acute HEV infection (*n* = 440), acute HEV‐4 infection (*n* = 50), HEV PCR‐positive (*n* = 217) and recovered individuals (*n* = 156), respectively. Although only one HEV serotype has been described to date [22], our study design ensured that all clinically relevant HEV genotypes (HEV‐1, ‐3 and ‐4) were assessed by including samples collected from Europe (Germany, Spain and Austria, HEV‐3), East Asia (China, HEV‐4), and South and South East Asia (Bangladesh and Vietnam, HEV‐1). All four assays detected anti‐HEV antibodies, irrespective of the genotype.

Moreover, the diagnostic sensitivity was also determined in a seroconversion context. For this purpose, nine commercially available seroconversion panels were tested, consisting of 8–12 samples per panel and spanning periods of 47–133 days. In most sample series, seroconversion was consistently detected by all four assays. However, IgG seroconversion in Panels C and G was detected 4–6 weeks and 1 week earlier, respectively, by Elecsys Anti‐HEV IgG than the comparator assays. In these respective panel samples, IgM was not detected with two out of four of the assays, which may indicate that HEV re‐infection has occurred, as shown in re‐infection experiments in rhesus macaques [23]. This would not be improbable, as it is estimated that approximately 12.5% of the world's population has already had contact with HEV at least once in their lives [1]. As anti‐HEV antibody levels generally decrease in the years following initial infection, and presumably fall to a level that is no longer protective, a large number of individuals may become susceptible to re‐infection with HEV [24, 25]. Taken together, these results indicate that the Elecsys assay is more sensitive in detecting anti‐HEV IgG than the comparator assays used in this study.

The relative specificity was assessed using a majority approach, whereby samples were considered negative if at least two of the three comparator HEV assays were negative. The relative specificity of the Elecsys Anti‐HEV IgM assay was 99.6%, 98.9% and 100% in blood donor samples (*n* = 5040), routine samples (*n* = 2427) and samples from pregnant women (*n* = 544), respectively. Considering the four assays are based on different detection technologies and/or use independent capture antigens, this indicates the reliability of anti‐HEV IgM detection by all four assays. It is noteworthy in this context that anti‐HEV IgM is not interpreted exclusively as a marker of acute HEV infection, since anti‐HEV IgM can persist for several months to years after infection [26, 27, 28, 29, 30]. The relative specificity of the Elecsys Anti‐HEV IgG assay for anti‐HEV IgG using a majority approach was remarkably lower, with 92.1%, 81.2% and 95.4% in blood donor samples (*n* = 5040), routine diagnostic samples (*n* = 2427) and samples from pregnant women (*n* = 544), respectively. This prompted further analysis of samples with discrepant results (i.e., those that were Elecsys Anti‐HEV IgG‐positive but majority‐negative with the comparator assays). Using a different HEV antigen, 97.7% (680/696) of the samples with discrepant results were neutralised, thus independently confirming the presence of anti‐HEV‐specific antibodies. This indicates that the majority consensus approach, as determined by the comparator assays, probably led to false‐negative results for these samples. Adjusting for these discrepant samples resulted in high relative specificities for the Elecsys Anti‐HEV IgG assay of 99.9%, 99.4% and 99.8% for blood donor samples, routine diagnostic samples and samples from pregnant women, respectively.

Previous comparative studies have found marked differences in the diagnostic performance between different HEV antibody detection assays [16, 31, 32, 33]. For example, in one study of 200 samples collected from healthy healthcare workers in Germany, anti‐HEV IgG was detected in 4.5%, 29.5% and 18.0% of the subjects with the Genelabs Technologies ELISA, the Wantai enzyme immunoassay and the Mikrogen *recom*Line immunoblot. Among 30 samples from patients with acute HEV infection, 83.3%, 100% and 96.7%, respectively, were anti‐HEV IgG‐positive using the same assays [16]. In an Australian study, among 194 samples that tested anti‐HEV IgG‐positive with the Wantai assay, only 47% and 65%, respectively, tested positive with the MP Diagnostics IgG and MP Diagnostics total HEV antibody assays [32]. Considering these results from previous studies, the new Roche Elecsys Anti‐HEV IgG assay seems to have a markedly higher sensitivity than comparator assays for detecting previous HEV infections and lower IgG concentrations than comparator assays.

In the absence of a ‘gold standard’ for HEV IgG and IgM, the choice of comparator assays and result definitions (majority or consensus) can influence sensitivity and specificity estimates. To address this potential bias, an overall percentage agreement analysis was performed (Figure 2; individual results provided in Figure S1), which demonstrated high concordance and overlapping confidence intervals across cohorts, confirming the robustness of our findings.

A limitation of the study is that the widely used Diasorin assay (Liaison Murex anti‐HEV IgG and IgM, DiaSorin, Saluggia, Italy) was not included as a comparator assay for the detection of anti‐HEV IgG/IgM; Diasorin HEV assays were not available when the design of the multicentre study was finalised. Further studies comparing the performance of the Elecsys assay (IgG and IgM) to the automated chemiluminescence assay from Diasorin are warranted to ascertain the potential merits of this method.

HEV represents a global public health issue, with HEV‐1 and ‐2 causing large outbreaks in subtropical countries, while HEV‐4 is endemic in Asia and HEV‐3 is endemic worldwide [1, 34, 35]. Additionally, rat HEV also poses a risk of human infections, with 37 cases documented so far [11]. As the clinical presentation of HEV infection is indistinguishable from other forms of hepatitis [15], reliable serologic assays are essential tools for diagnosis that can be amended by adsorption methods and diagnostic algorithms [36]. As anti‐rat HEV antibodies may be partially detected by HEV‐specific immunoassays and vice versa [37, 38], molecular approaches for the detection of viral RNA remain essential for definitive discrimination. Interestingly, partial cross‐protection against rat HEV has been observed in Mongolian gerbils initially infected with HEV‐1 [39].

Awareness and incident cases of acute HEV have increased in recent years [25, 34, 40]. Increased demand for HEV testing can be met using automated assay systems that facilitate the processing of higher volumes of samples; however, there are only a limited number of automated systems currently available for the detection of anti‐HEV antibodies. The Elecsys assays are automated and conducted on widely available analysis platforms that facilitate on‐demand processing of large sample volumes, with short time‐to‐result and reliable detection of anti‐HEV IgM and anti‐HEV IgG.

## 5 Conclusions

In summary, our study shows that the novel automated Elecsys Anti‐HEV IgG and IgM detection assays have good overall analytic performance and higher sensitivity for low anti‐HEV IgG concentrations than the other commercial assays studied. Our findings indicate that these assays can be reliably used in a diagnostic context for the serologic detection of recent or past infections with HEV.

## Author Contributions

Study concept and design: T.S., K.K., J.P. and C.V. Acquisition of data: M.S., T.S., K.K., J.L., K.M.M.‐S., A.M., J.P., H.S., Q.X., J.W. and M.L. Statistical analysis: L.S.P., T.S., K.K., J.P. and M.L. Interpretation of data: M.S., L.S.P., T.S., K.K., J.P., C.V., J.W. and M.L. Drafting manuscript: M.S., L.S.P., T.S., K.K., J.P., C.V., J.W. and M.L. Critical revisions of the manuscript for important intellectual content: L.S.P., T.S., J.L., K.K., J.P., Q.X., C.V., J.W. and M.L.

## Funding

This work was supported by Roche Diagnostics GmbH Penzberg, Germany.

## Disclosure

Disclaimer: Elecsys Anti‐HEV IgG and Elecsys Anti‐HEV IgM assays: For Research Use Only in the United States and not for diagnostic procedures.

## Conflicts of Interest

M.S.: received a lecture fee from Mikrogen GmbH; LSP: received speakers' honoraria from Roche Diagnostics, and is funded by the “Deutsche Forschungsgemeinschaft” (German Research Foundation (DFG), iDfellows: Hamburg Clinician Scientist Programme in Infectious Diseases, project number: 493624519); T.S.: Roche employee; K.K.: Roche employee; J.P.: Roche employee; C.V.: Roche employee; M.L.: received travel expenses and speakers' honoraria from Roche Diagnostics. J.L., K.M.M.‐S., A.M., H.S., Q.X. snd J.W.: no conflicts to disclose.

## Supporting information


**Data S1:** liv70480‐sup‐0001‐supinfo.docx.

## Data Availability

The data that support the findings of this study are available on request from the corresponding author. The data are not publicly available due to privacy or ethical restrictions.
