# Supplementary material for: Performance of Elecsys Anti‐HEV IgG and IgM Assays for the Detection of Acute, Recent or Past Hepatitis E Virus Infection
Source: Liver Int. 2025 Dec 15;46(1):e70480. doi: 10.1111/liv.70480 (PMC12704027; doi:10.1111/liv.70480)
Supplement: Supplementary file 1 — Data S1: liv70480‐sup‐0001‐supinfo.docx. [file LIV-46-0-s001.docx]

**Performance of Elecsys Anti-HEV IgG and IgM assays for the detection of acute, recent, or past hepatitis E virus infection**

**Supplementary Materials**

**Contents**

[Neutralisation assay 2](#_Toc210297493)

[Table S1: Overview of study sites 4](#_Toc210297494)

[Table S2: Study sites and samples used for the sensitivity and specificity cohorts 5](#_Toc210297495)

[Table S3: Elecsys and comparator assays assay information 7](#_Toc210297496)

[Table S4: Sensitivity of Elecsys Anti-HEV IgG assay and comparator assays for seroconversion sensitivity and titre development 9](#_Toc210297497)

[Fig. S1: OPA between the Elecsys Anti-HEV IgM or the Elecsys Anti-HEV IgG assay and comparator assays for the sensitivity and specificity cohorts 10](#_Toc210297498)

# **Neutralisation assay**

**Principle:** the neutralisation protocol is based on the addition of recombinant HEV antigen, capsid protein (ORF2) expressed in Hansenula polymorpha (yeast), to a sample under investigation. This antigen is referred to as neutralising HEV-antigen (nHEV-antigen). In the presence of anti-HEV IgG in a given sample, the added nHEV-antigen and the anti-HEV IgG form immune complexes. The nHEV-antigen-bound anti-HEV IgGs are no longer available for binding to the biotin- and ruthenium-labelled HEV antigen components of the Elecsys Anti-HEV IgG assay. Consequently, when the sample is

re-measured with the Elecsys Anti-HEV IgG assay, the detected concentration of anti-HEV IgG is lower compared to the sample before neutralisation, confirming the sample as true anti-HEV IgG positive. In the case of an interfering factor that causes unspecific signal generation in the absence of anti-HEV IgG, the addition of nHEV-antigen does not alter the Elecsys Anti-HEV IgG assay signal, confirming the sample as false positive. If the sample is anti-HEV IgG negative, the addition of nHEV-antigen has no effect on the measured signal as nHEV-antigen does not interact with the Elecsys Anti-HEV IgG components. The sample, thus, remains non-reactive with the Elecsys Anti-HEV IgG assay and is confirmed as true negative. This principle was also used by other studies to confirm the specificity of *Toxpoplasma gondii* IgG (Köhler et al., 2010) and *Trypanosoma cruzi* IgG (Chavez et al., 2018).

**Protocol:** a 30 µl volume of a 10x concentrated nHEV-antigen (HyTest Ltd, Finland) stock solution (50 U/ml neutralising activity; diluted in anti-HEV IgG negative human serum) was added to 270 µl sample volume to achieve a final concentration of 1x (5 U/ml nHEV-antigen neutralising activity). The IC50 (50% recovery of anti-HEV IgG reactive samples incubated with nHEV) is defined as 1 U/ml, and 5 U/ml of nHEV-antigen neutralising activity was selected as the standard concentration for the final neutralisation protocol. Similarly, anti-HEV IgG non-reactive serum (reference) is added to a second aliquot of the sample at one-tenth of its volume to account for the change in volume. After a minimum incubation period of 60 minutes at room temperature on a tilt/roller mixer (neutralisation process), the two aliquots (with and without nHEV-antigen) were measured using the Elecsys Anti-HEV IgG assay, and the percentage recovery was calculated as a measure of neutralisation. If the recovery was ≤50%, the sample was considered confirmed-reactive (i.e., a true positive result). If the recovery was >50%, the sample was considered false-reactive with the Elecsys Anti-HEV IgG assay.

**Validation:** for validation, a lot-to-lot comparison of three nHEV-antigen lots (HyTest Cat #8HEV3, lot IDs 16/04-8HEV3, 17/06-8HEV3, 21/02-8HEV3) was performed using a sample panel (n=146) that spanned the measuring range of the Elecsys Anti-HEV IgG assay (0.05–25 U/ml). Samples were processed with three standardised nHEV-antigen lots (validated for IC50/neutralising activity). A linear regression was achieved with slopes of 1.00 ± 0.05 and a y-intercept of 0.0 ± 0.05 U/ml. Precision was validated by splitting anti-HEV IgG reactive samples into 21 aliquots, with each aliquot fully processed according to the neutralisation protocol. Recovery after neutralisation was calculated for each processed aliquot, and the standard deviation and coefficient of variation (CV) were determined. A mean CV of less than 5% was achieved for recovery measurements.

The specificity of nHEV-antigen in binding only to anti-HEV IgG was assessed by processing serum samples (n=60) positive for antibodies against other infectious agents, including hepatitis A, hepatitis B, hepatitis C, Toxoplasma, Rubella, and Cytomegalovirus. These samples were re-measured with the respective Elecsys infectious disease assay, and the recovery for each parameter after neutralisation was found to be within 100 ± 20%. To evaluate possible differences between serum and plasma, matched serum-plasma sets (n=12) obtained from Elecsys Anti-HEV IgG reactive and non-reactive donors were processed according to the neutralisation protocol. Linear regression revealed slopes of 1.00 ± 0.05 and a y-intercept of 0.0 ± 0.05 U/ml. The neutralisation protocol was further verified using anti-HEV IgG positive and negative samples (n=38 and n=67 respectively). Positive samples were concordant with three competitor assays (Wantai, Mikrogen, bioMérieux) as well as the Elecsys Anti-HEV IgG assay. Likewise, negative samples exhibited concordant results across all four assays. The final neutralisation protocol was applied as described, and agreement rates with sample status (confirmed reactive or non-reactive) were determined.

# **Table S1: Overview of study sites**

| **Study site** | **Assays** | **Platform** |
| --- | --- | --- |
| **Synlab Labor München,** Zentrum Medizinisches Versorgungszentrum GbR, München, Germany | Elecsys^®^ Anti HEV IgM and IgG (Roche) | Cobas e 411 (Roche) |
|  | Wantai HEV IgM ELISA and IgG | BEPIII (Siemens) |
|  | *recom*Well HEV IgM and IgG (Mikrogen) | BEPIII (Siemens) |
|  | Vidas^®^ Anti-HEV IgM and IgG (Biomerieux) | Mini Vidas (Biomerieux) |
| **University Medical Center Regensburg**  Regensburg, Germany | Elecsys Anti HEV IgM and IgG | Cobas e 411 |
|  | Wantai HEV IgM ELISA and IgG | Tecan Infinite 50 |
|  | *recom*Well HEV IgM and IgG | Tecan Infinite 50 |
|  | Vidas Anti-HEV IgM and IgG | Mini-Vidas |
| **Labor Krone eGbR**,  Bad Salzuflen, Germany | Elecsys Anti HEV IgM and IgG | Cobas pro (e 801; Roche) |
|  | Wantai HEV IgM ELISA and IgG | Euroimmun Workstation |
|  | *recom*Well HEV IgM and IgG | Euroimmun Workstation |
|  | Vidas Anti-HEV IgM and IgG | Mini-Vidas |
| **University Hospital Eppendorf,**  Hamburg, Germany | Elecsys Anti HEV IgM and IgG | Cobas e 411 |
|  | Wantai HEV IgM ELISA and IgG | Euroimmun Analyzer I |
|  | *recom*Well HEV IgM and IgG | Euroimmun Analyzer I |
|  | Vidas Anti-HEV IgM and IgG | Vidas testing at UKR |
| **Tirol Kliniken Landeskrankenhaus,**  Innsbruck,  Austria | Elecsys Anti HEV IgM and IgG | Cobas e 601 (Roche) |
|  | Wantai HEV IgM ELISA and IgG | ETIMAX 3000 (Diasorin) |
|  | *recom*Well HEV IgM and IgG | ETIMAX 3000 |
|  | Vidas Anti-HEV IgM and IgG | Vidas testing at Labor Krone |
| **Shanghai Ruijin Hospital**,  Shanghai, China | Elecsys Anti HEV IgM and IgG | Cobas e 411 |
|  | Wantai HEV IgM ELISA and IgG | N/A^a^ |
|  | *recom*Well HEV IgM and IgG | N/A^a^ |
|  | Wantai InnoDx HEV IgM and IgG | Caris 200 (Wantai) |

^a^Wantai HEV IgM ELISA and IgG and *recom*Well HEV IgM and IgG assays were performed manually at this site.

# **Table S2: Study sites and samples used for the sensitivity and specificity cohorts**

|  | **Synlab Labor** | **University Medical Center Regensburg** | **Labor Krone** | **University Hospital Eppendorf** | **Tirol Kliniken Landeskrankenhaus** | **Shanghai Ruijin Hospital** |
| --- | --- | --- | --- | --- | --- | --- |
| **Ethics approval** | Waiver from Bavarian Landesaerztekammer | EC waiver from 10-Dec-2021 | EC waiver from 7-Apr-2022 | EC waiver from 7-Jun-2022 | EC approval from 23-Mar-2022; AGES approval from 30-Mar-2022 | EC approval from 01-Aug-2022; HGRAC approval from 09-Oct-2022 |
| **Sensitivity cohort** | No samples tested | 102 presumed acute HEV-3 samples | Presumed acute samples:   - 132 HEV-1 positive from Bangladesh^a,b^ - 56 HEV-1 positive from Vietnam^c^ - 100 HEV-3 positive from Spain^d^ - 112 HEV-RNA PCR positive samples^e^ | 50 HEV-3 presumed acute samples  105 HEV-RNA PCR positive samples  156 HEV Recovered | No samples tested | 50 HEV-4 presumed acute samples |
| **Specificity cohort** | 1,000 routine samples | 400 routine samples | 1,027 routine samples  544 pregnant women samples | No samples tested | 5,040 blood donor samples | No samples tested |

Commercial vendors: ^a^Slieagen; ^b^Simon Lytton; ^c^Discovery; ^d^BioIVT; ^e^Trina.

HEV, hepatitis E virus

# **Table S3: Elecsys and comparator assays assay information**

| **Anti HEV IgM** | | | | |
| --- | --- | --- | --- | --- |
|  | **Elecsys Anti-HEV IgM** | ***recom*Well HEV IgM** | **Wantai HEV IgM ELISA** | **VIDAS Anti-HEV IgM** |
| Manufacturer | Roche | Mikrogen | Wantai | bioMérieux |
| Format | Qualitative | Qualitative or semi-quantitative | Qualitative | Qualitative |
| Assay principle | 1-step ECLIA | ELISA | ELISA | 2-step ELFA |
| Automated | Yes | Possible | Possible | Yes |
| Antigens | ORF2 (HEV-1, HEV-3) | ORF2 (HEV-1, HEV-3) | ORF2 (HEV-1) | ORF2/ORF3 (HEV-1) |
| Specimen type | Serum; Li‑heparin, Na‑heparin, K_2_‑EDTA, K_3_‑EDTA, Na‑citrate plasma | serum; EDTA, citrate, heparin plasma; CPD | serum; EDTA, heparin, Na-citrate plasma | serum; EDTA-, Li-heparin |
| Sample volume | 10 µL^a^ or 6 µL^b^ | 10 µL | 10 µL | 100 µL |
| Reaction time | 18 min | ~120 min | ~90 min | 40 min |

| **Anti HEV IgG** | | | | |
| --- | --- | --- | --- | --- |
|  | **Elecsys Anti-HEV IgG** | **recomWell HEV IgG** | **Wantai HEV IgG ELISA** | **VIDAS Anti-HEV IgG** |
| Manufacturer | Roche | Mikrogen | Wantai | bioMérieux |
| Format | Quantitative and qualitative | Qualitative or semi-quantitative | Qualitative | Quantitative |
| Linear range | 0.05–25 U/mL | n/a | n/a | 0.1–10 U/mL |
| Assay principle | 1-step ECLIA | ELISA | 2-step ELISA | 2-step ELFA |
| Automated | Yes | Possible | Possible | Yes |
| Antigens used  for anti-HEV detection | ORF2 (HEV-1, HEV-3) | ORF2 (HEV-1, HEV-3) | ORF2 (HEV-1) | ORF2/ORF3 (HEV-1) |
| Specimen type | Serum, Li‑heparin, Na‑heparin, K_2_/K_3_‑EDTA, Na‑citrate plasma | serum; EDTA, citrate,  heparin plasma; CPD | serum; EDTA, heparin, Na-citrate plasma | serum; EDTA-, Li-heparin |
| Sample volume | 20 µL^a^ or 12 µL^b^ | 10 µL | 10 µL | 100 µL |
| Reaction time | 18 min | ~120 min | ~90 min / 29 min | 40 min |

^a^Sample volume for Cobas e 402 and e 801. ^b^Sample volume for Cobas e 411, e 601 and
e 602.

ECLIA, electrochemiluminescence assay; HEV, hepatitis E virus; ORF, open reading frame.

# **Table S4: Sensitivity of Elecsys Anti-HEV IgG assay and comparator assays for seroconversion sensitivity and titre development**

| **Panel** | **Elecsys Anti-HEV IgG, U/mL (days since 1^st^ bleed)** | | | | | |
| --- | --- | --- | --- | --- | --- | --- |
|  | **Last Elecsys Anti-HEV IgG non-reactive** | **Elecsys Anti-HEV IgG -reactive bleed** | | | | |
|  |  | **1^st^** | **2^nd^** | **3^rd^** | **4^th^** | **5^th^** |
| Panel A  SCP-HEV-001b | 0.103  (38 d) | 1.89  (41 d) | 5.39  (46 d) | 8.97  (49 d) | 16.2  (53 d) | 58.2  (77 d) |
| Panel B  SCP-HEV-002b | 0.000173  (25 d) | 0.262  (35 d) | 6.00  (42 d) | 31.4  (68 d) | 60.4  (84 d) | n.a. |
| Panel C  SCP-HEV-003a | n.a. | 0.315  (0 d) | 0.291  (6 d) | 7.15  (21 d) | 13.0  (28 d) | 101  (42 d) |
| Panel D  SCP-HEV-004a | n.a. | 4.80  (0 d) | 18.9  (11 d) | 15.7  (14 d) | 17.5  (18 d) | 22.2  (22 d) |
| Panel E  SCP-HEV-005b | 0.0179  (21 d) | 3.61  (28 d) | 4.10  (35 d) | 7.40  (42 d) | 13.0  (49 d) | 55.9  (63 d) |
| Panel F  SCP-HEV-006b | 0.0643  (46 d) | 1.25  (50 d) | 0.907  (56 d) | 23.7  (105 d) | 24.1  (108 d) | 26.7  (112 d) |
| Panel G  SCP-HEV-007a | 0.0579  (28 d) | 0.298  (32 d) | 0.604  (39 d) | 2.71  (46 d) | 4.29  (52 d) | 6.86  (59 d) |
| Panel H  SCP-HEV-008a | 0.00130  (0 d) | 15.8  (46 d) | 19.0  (49 d) | 22.8  (54 d) | 33.9  (61 d) | 33.2  (64 d) |
| Panel I  SCP-HEV-009a | 0.000430  (21 d) | 27.2  (59 d) | 24.4  (63 d) | 46.5  (80 d) | 38.7  (84 d) | 34.8  (105 d) |

d, day; HEV, hepatitis E virus.

# **Fig. S1: OPA between the Elecsys Anti-HEV IgM or the Elecsys Anti-HEV IgG assay and comparator assays for the sensitivity and specificity cohorts**

**
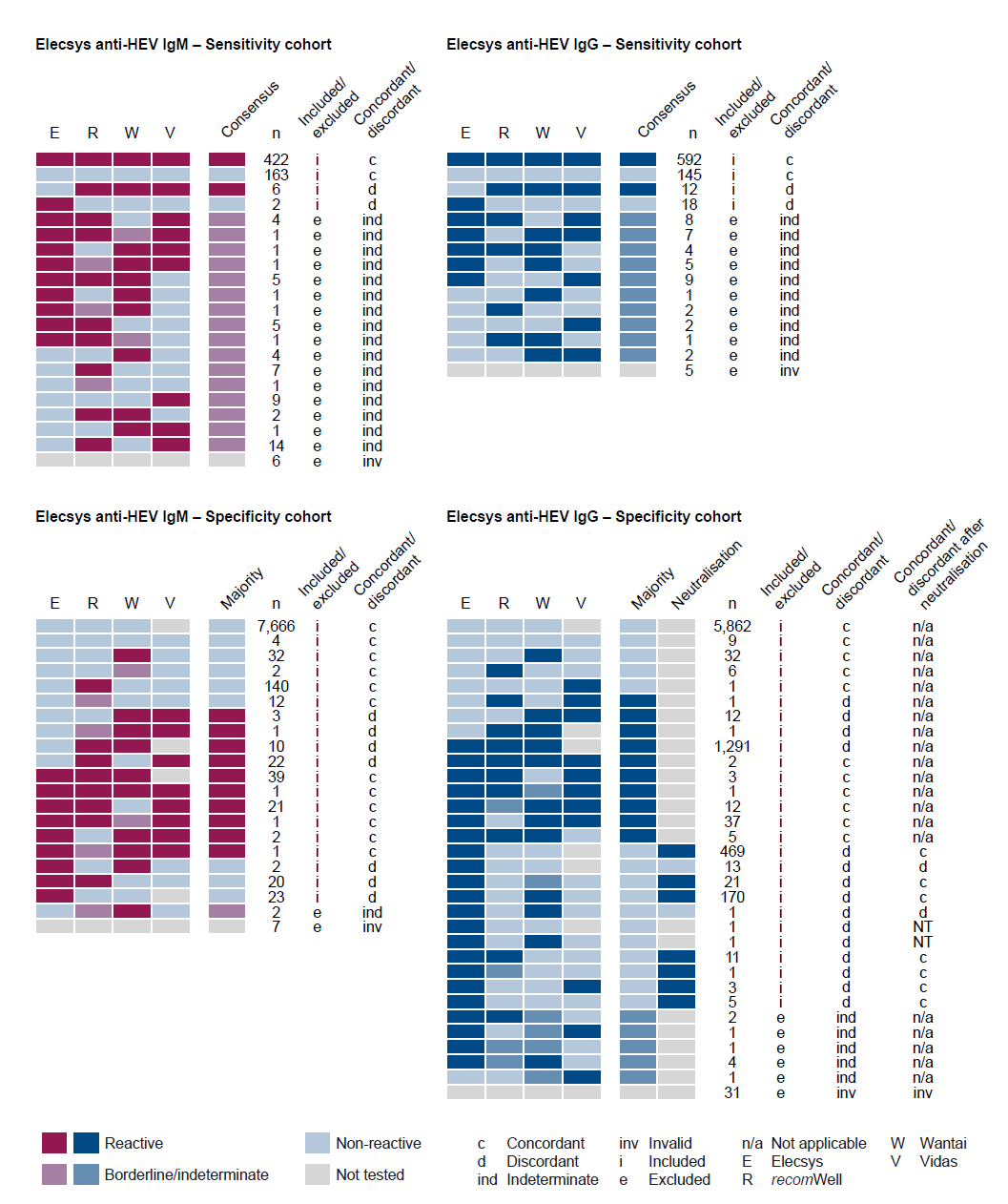
**

C, concordant; d, discordant; E, Elecsys; e, excluded; HEV, hepatitis E virus; i, included; ind, indeterminate; inv, invalid; n/a not applicable; NT, not tested; OPA, overall percentage agreement; R, *recom*Well; V, Vidas; W, Wantai.
